# Electroconvulsive therapy in children and adolescents: a case report

**DOI:** 10.1192/j.eurpsy.2022.1103

**Published:** 2022-09-01

**Authors:** L. Mallol Castaño, P. Del Sol Calderón, R. Paricio Del Castillo

**Affiliations:** Hospital Universitario Puerta de Hierro, Psiquiatría Infanto-juvenil, Madrid, Spain

**Keywords:** Electroconvulsive therapy, PSYCHOTIC DISORDERS, mood disorders, children and adolescentes

## Abstract

**Introduction:**

Electroconvulsive therapy is a proven treatment for mood and psychotic disorders in adult patients. It is estimated that in children and adolescents this type of therapy is underutilised despite the fact that the most recent studies have supported the success of ECT in these patients. A case is described of a 15-year-old male patient diagnosed with psychotic disorder who was previously treated with several antipsychotics, including clozapine, and finally treated with electroconvulsive therapy.

**Objectives:**

Review of the clinical indications of electroconvulsive therapy in children and adolescents with psychotic or mood disorders through a clinical case of a patient admitted to a Psychiatric Short Stay Unit

**Methods:**

Detailed psychopathological description of the case as well as the treatments used (psychotropic drugs and electroconvulsive therapy).

**Results:**

After the administration of electroconvulsive therapy, an improvement in both positive and negative psychotic symptomatology was observed, with a decrease in soliloquies and an improvement in affective flattening.

**Conclusions:**

Electroconvulsive therapy is an effective treatment in adolescent patients with psychotic and mood disorders, which should be considered as indicated as an effective treatment.

**Disclosure:**

No significant relationships.

